# A Novel Demodulation Analysis Technique for Bearing Fault Diagnosis via Energy Separation and Local Low-Rank Matrix Approximation

**DOI:** 10.3390/s19173755

**Published:** 2019-08-30

**Authors:** Yong Lv, Mao Ge, Yi Zhang, Cancan Yi, Yubo Ma

**Affiliations:** 1 Key Laboratory of Metallurgical Equipment and Control Technology, Wuhan University of Science and Technology, Ministry of Education, Wuhan 430081, China; 2 Hubei Key Laboratory of Mechanical Transmission and Manufacturing Engineering, Wuhan University of Science and Technology, Wuhan 430081, China

**Keywords:** bearing fault diagnosis, modulation signal, demodulation analysis, teager energy operator, local low-rank matrix approximation

## Abstract

Bearing fault diagnosis is of utmost importance in the maintenance of mechanical equipment. The collected fault vibration signal generally presents a modulated nature due to the special structure and dynamic characteristics of the bearings. This paper introduces a novel demodulation analysis technique via energy separation and local low-rank matrix approximation (LLORMA) to address this type of signal. The amplitude envelope and instantaneous frequency of the signal can be calculated via an energy separation algorithm based on the Teager energy operator. We can confirm the bearing faults by comparing the peak frequencies of the Fourier spectrum of the amplitude envelope and instantaneous frequency with the theoretical bearing fault-related frequencies. However, this algorithm is only suitable for handling single-component signals. In addition, the powerful background noise has a serious effect on the demodulation results. To tackle these problems, a new signal decomposition method based on LLORMA is proposed to decompose the signal into several single-components and eliminate the noise simultaneously. After that, the single-component signal representing the fault characteristics can be identified via the high frequency feature of the modulated signal. The analysis of the simulated signal and the bearing outer race fault signal collected from a bearing-gear fault test rig indicate that the proposed technique has an excellent diagnostic performance for bearing fault signals.

## 1. Introduction

As the crucial component that bears most of the load in rotating machinery, the health status of bearings can directly influence the working performance of the whole mechanical system [1,2]. Due to difficult working conditions, such as overload, high temperature, corrosion, and so on, bearings are prone to faults, which can lead to downtime and even huge economic losses [3]. Therefore, bearing fault diagnosis is an important research topic [4].

Bearing faults mainly appear in the contact surface, such as wear or cracks on the inner race, outer race, and rolling element. To deal with the bearing faults mentioned above, Scholars have proposed many signal-processing methods. Smith et al. [5] proposed an optimized spectral kurtosis method to identify bearing faults under electromagnetic interference. The Wigner–Ville distribution (WVD) [6] can effectively extract the characteristic information of non-stationary signals from the time–frequency plane. Ming et al. [7] extracted the weak fault feature of the rolling bearing signal though a method based on the cyclic Wiener filter and envelope spectrum. However, the anti-noise performance of WVD is insufficient, and there are cross interference terms when processing multi-component signals. Signal processing methods based on wavelet transform (WT) can achieve a high signal-to-noise ratio (SNR) and decompose any signal into low-frequency components and high-frequency components. Wang et al. [8] proposed a novel adaptive wavelet stripping algorithm for extracting the transients caused by localized bearing faults. Zhang et al. [9] used wavelet packet decomposition and the random forest method to realize the fault diagnosis of a rolling bearing. However, the choice of wavelet basis function and thresholds has an important effect on the diagnosis results of these methods. Daubechies et al. [10] proposed a new synchrosqueezed wavelet transform method to extract ridges representing characteristic signal information from the time–frequency plane of the signal. Hu et al. [11] introduced this method into the field of mechanical equipment fault diagnosis and successfully extracted the fault characteristics of bearings and gears. Nevertheless, the existence of noise will cause blurring of the time–frequency plane, which makes it difficult to extract the correct ridge. Besides, it will produce ridge crossing in the time–frequency plane for multi-component signals. These studies have considerably enriched the literature related to bearing fault diagnosis.

However, because of the special structure and dynamic characteristics of bearings, periodic impulses will be produced when local faults occur on the surface of a bearing element. Due to the short duration of these pulses and the wide bandwidth of their energy distribution, in the vibration signal spectrum composed of these pulses, a high energy resonance frequency band around the system resonance frequency is formed, which leads to amplitude modulation (AM) and frequency modulation (FM), resulting in an increase in spectral complexity. By contrast, the energy in the low frequency band of the signal spectrum where the fault-related frequencies represented by the fault characteristic frequency and its first few harmonics are located is extremely weak [12]. In general, the system resonance frequency is the signal carrier frequency, while the fault characteristic frequency of the fault element or its harmonics represents the modulation frequency [13]. The crux of fault diagnosis is differentiating these fault-related frequencies from the signal spectrum. Since the spectral characteristics of the AM component and FM component are relatively simple and closely related to the fault-related frequencies, the bearing fault information can be revealed in the spectrum of the AM component and the instantaneous frequency spectrum of the FM component. Hence, we can use a demodulation method to separate these low-frequency modulation components from the high-frequency carrier signal, the core of which is to estimate the amplitude envelope or instantaneous frequency of the signal [14].

High-frequency resonance (HFR) [15] is a widely used amplitude demodulation method in industry. This method firstly carries out bandpass filtering near the resonance frequency and then performs amplitude demodulation (AD) to obtain a signal. It should be noted that most of the energy of the acquired signal is concentrated near the fault or its harmonic frequencies. Osman et al. [16] proposed a leakage-free resonance sparse decomposition method to detect the bearing fault in a gearbox. The Hilbert transform [17] can effectively estimate the amplitude envelope and instantaneous frequency of a signal. However, the major challenge in the practical application of these traditional demodulation methods is the selection of a resonance frequency and bandpass filter bandwidth. In particular, the estimation of the resonance frequency directly determines the final demodulation result.

In contrast, as a completely data-driven demodulation algorithm without any parameter estimation, the energy separation algorithm based on the Teager energy operator can effectively calculate the energy required to generate a signal by using a non-linear operator [18,19]. By separating this non-linear operator, the amplitude envelope and instantaneous frequency of the signal can be obtained. Nevertheless, this algorithm can only handle single-component modulated signals. In addition to the bearing fault characteristic vibration signal, the measured signal contains the interference vibration caused by other mechanical components, such as gear meshing [14]. Furthermore, massive and powerful background noise has a strong effect on the energy estimation results. So, suppressing the background noise and extracting the fault characteristic signal is critical. The local mean decomposition (LMD) [3] method, consisting of an amplitude envelope signal and a purely frequency-modulated signal, can decompose any signal into a series of product functions (PFs) with physical meaning. For the fault diagnosis in the machinery field, researchers have proposed several joint fault diagnosis techniques based on the energy separation algorithm and LMD or its improved version [20,21,22]. Nevertheless, the LMD and its improved version not only suffer from modal aliasing but are also sensitive to noise.

Generally, the attractor in high-dimensional phase space formed by vibration signal reconstruction can demonstrate the dynamic properties of the working conditions of different components in the original system well [23,24]. Thus, the high-dimensional phase space signal processing technique can provide a feasible method for fault feature extraction. As a well-known method for addressing one-dimensional time series, singular spectrum analysis (SSA) [25] can decompose the signal attractor matrix into different singular subspaces which can be distinguished into different components, such as the fault characteristic component and the unwanted interfering components or noise. However, the selection of a singular value is a non-deterministic polynomial (NP) hard problem, and especially in the presence of noise, the singular value of the attractor matrix will deviate from the true value [26]. The proposed robust principal component analysis (RPCA) [27,28] technique can eliminate the deviation of the singular value by constructing penalty function regularization and extracting the useful component in the attractor matrix via low-rank matrix approximation. RPCA can effectively suppress the noise in the vibration signal, but it cannot decompose a multi-component signal into single components. Recently, Lee et al. [29] proposed an interesting local low-rank matrix approximation (LLORMA) method for the data completion problem. Instead of the principle in RPCA that the matrix is approximated by a single low-rank subspace, LLORMA supports the decomposition of a matrix formed by a linear combination of multiple low-rank subspaces into their respective single subspaces, and the matrix does not require a low-rank structure. Therefore, there is potential to decompose the multi-component bearing signal into single components by LLORMA.

Inspired by the above analysis, this paper introduces a novel demodulation technique via energy separation and LLORMA for bearing fault diagnosis. Firstly, we propose a new LLORMA-based signal decomposition method to decompose the signal into several single components and meanwhile eliminate the noise. Our method indicates that the signal attractor matrix behaves as the sum of a low-rank component and noise component in the phase space of the weighted matrix associated with the selected anchor point. Both of these components can be extracted by solving a convex optimization problem about the joint minimization of the matrix nuclear norm and the penalty function regularization [30,31]. Then, the low-rank matrices representing the fault characteristic component can be identified through the high-frequency feature of the instantaneous frequency of the modulated signal. After that, these low-rank matrices are combined into a one-dimensional signal representing the fault characteristics via the linear regression model [32] and inverse phase space reconstruction. Next, the amplitude envelope and instantaneous frequency of the extracted one-dimensional signal are estimated by the energy separation algorithm, and the Fourier transform is applied to them. Finally, we can confirm the presence of a bearing fault by comparing the peak frequencies of the envelope spectrum and instantaneous frequency spectrum with the theoretical bearing-fault-related frequencies. The analysis of the simulated signal and the bearing outer race fault signal collected from a bearing-gear fault test rig demonstrate that the proposed technique can detect and locate the bearing fault accurately.

The rest of the paper is organized as follows: Section 2 introduces the signal model of bearing fault vibration and the theory of the energy separation algorithm. The proposed novel demodulation analysis technique is presented in Section 3. The analyses of the simulated signal and the collected bearing fault signal are described in Section 4. Section 5 draws the conclusions.

## 2. Bearing Fault Vibration Signal Model and Demodulation Analysis through the Energy Separation Algorithm

### 2.1. Vibration Signal Model of the Bearing Fault

The collected characteristic vibration signal of the bearing fault can be represented by a series of pulsed excitation signals [12,33,34,35]:

(1) $$x(t)=c(t)\sum_{i=0}^{I}a_{i}(t)\cos(2\pi f_{n}t-b_{i}(t)+\varphi_{i})$$

where c(t) represents the modulation effect produced by the vibration propagation path; $a_{i}(t)$ and $b_{i}(t)$ represent the AM component and FM component, respectively; $f_{n}$ and $\varphi_{i}$ represent the system resonance frequency and initial phase, respectively.

$a_{i}(t)$ and $b_{i}(t)$ can be written as

(2) $$a_{i}(t)=A_{i}e^{-\beta (t-iT_{p}-\tau_{i})}u(t-iT_{p}-\tau_{i})$$

(3) $$b_{i}(t)=\sum_{l=1}^{L}B_{il}\sin(2\pi lf_{c}t+\varphi_{il})$$

where β is the system attenuation coefficient, $f_{c}$ is the fault characteristic frequency, $T_{p}=1/f_{c}$ is the time period corresponding to $f_{c}$, $\tau_{i}$ represents the random slip of the *i*-th pulse, u(t) is a unit step function, $\varphi_{il}$ is the initial phase in $b_{i}(t)$, $A_{i}$ and $B_{il}$ are the amplitudes of $a_{i}(t)$ and $b_{i}(t)$, respectively.

A vibration sensor is usually installed in the position of the bearing seat fixed with the outer race. When faults occur in the rolling element or the inner race, the vibration propagation path from them to the sensor usually produces an AM effect on the signal due to these two parts rotating relative to the outer race. Thus, c(t) can be expressed as

(4) $$c(t)=C[1+\cos(2\pi f_{r}t)]$$

where $f_{r}$ is the rotation frequency of the shaft, and C is a constant.

When a fault occurs in the outer race, since the vibration propagation path is fixed, the propagation path only affects the value of the signal amplitude:

(5) c(t)=C

**Figure 1:**
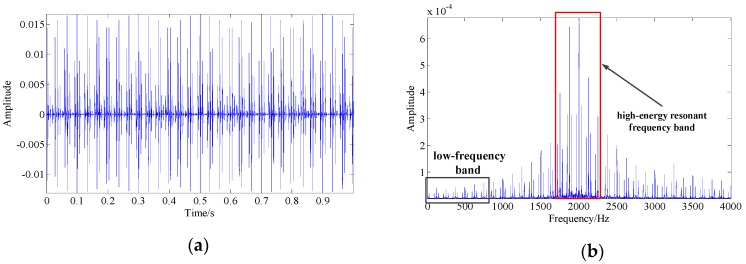

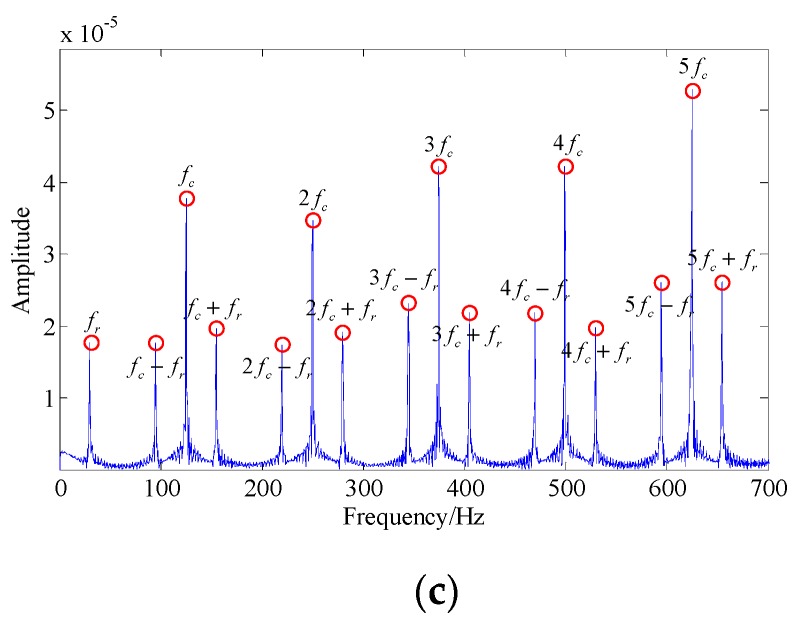
The simulated fault characteristic signal of the bearing inner race with a system resonance frequency of $f_{n}=2000$ Hz, a fault characteristic frequency of $f_{c}=125$ Hz, and a shaft rotation frequency of $f_{r}=30$ Hz: (**a**) simulated signal waveform; (**b**) simulated signal spectrum; (**c**) the low-frequency band (0–800 Hz) of the simulated signal spectrum.

A common method used to diagnosis the bearing fault is to detect the appearance of the peaks at the location of the fault-related frequencies in the signal spectrum. Figure 1 shows the waveform and spectrum of a simulated fault characteristic signal of the bearing inner race. It can be observed that the AM/FM effects increase the spectral complexity of the signal, and there is a high-energy resonance frequency band around the system’s resonance frequency ($f_{n}=2000$ Hz) in the spectrum. In contrast, the energy in the low-frequency band is shown in Figure 1c, where the fault-related frequency (the rotation frequency of the shaft $f_{r}$, the fault characteristic frequency $f_{c}$, its first few harmonics $2f_{c}$–$5f_{c}$ and their combination $f_{c}\pm f_{r}$–$5f_{c}\pm f_{r}$) locations are extremely weak and are easily drowned out by noise and interference, resulting in difficulty detecting and locating the bearing fault.

### 2.2. Demodulation Analysis through the Energy Separation Algorithm

As the spectral characteristics of AM and FM components are relatively simple and related to the bearing fault characteristic information, using the demodulation method, these two low-frequency modulation components can be separated from the high frequency carrier signal, achieving the aim of fault diagnosis. The core problem of extracting these two components is the difficulty in estimating the amplitude envelope and instantaneous frequency of the signal. This is different from the traditional demodulation method, which requires the selection of the center frequency and bandwidth of the bandpass filter, that is, the estimation of the resonance frequency is very important for the demodulation results. The energy separation method based on the Teager energy operator is a completely data-driven demodulation method without any parameter estimation, which can estimate the amplitude envelope and instantaneous frequency of an AM/FM signal through a nonlinear operator.

#### 2.2.1. Energy Separation Algorithm based on the Teager Energy Operator

For a given FM/AM signal x(t) with a time-varying amplitude a(t) and phase φ(t) [14,18]:

(6) x(t)=a(t)cos(φ(t))

Its instantaneous frequency can be calculated using

(7) $$f(t)=\frac{1}{2\pi }\dot{\varphi }(t)=\frac{1}{2\pi }d\varphi (t)/dt$$

The Teager energy operator of Equation (6) is defined as

(8) $$\psi (x(t))={\left[\dot{x}(t)\right]}^{2}-x(t)\ddot{x}(t)$$

where $\dot{x}(t)$ and $\ddot{x}(t)$ are the first and second derivatives of x(t). Equation (8) can be expanded to

(9) $$\psi (x(t))={\left[a(t)\dot{\varphi }(t)\right]}^{2}+a^{2}(t)\ddot{\varphi }(t)\sin[2\varphi (t)]/2+\cos^{2}[\varphi (t)]\psi (a(t))$$

In general, compared with the carrier signal, the time-varying rate of the modulation signal is slow, that is, a(t) and f(t) vary in a slow manner. So, they can be regarded as approximate constants: ψ(a(t))≈0, $\ddot{\varphi }(t)\approx 0$. Thus, Equation (9) becomes

(10) $$\psi (x(t))\approx a^{2}(t){\dot{\varphi }}^{2}(t)=4\pi^{2}a^{2}(t)w^{2}(t)$$

Similarly, the Teager energy operator of the first derivative of x(t) can be expressed as

(11) $$\psi (\dot{x}(t))\approx 16\pi^{4}a^{2}(t)w^{4}(t)$$

By simultaneously solving Equations (10) and (11), the absolute value of amplitude envelope and the instantaneous frequency of the AM/FM signal can be obtained:

(12) $$\left|a(t)\right|\approx \frac{\psi (x(t))}{\sqrt{\psi (\dot{x}(t))}}$$

(13) $$w(t)=\frac{1}{2\pi }\sqrt{\frac{\psi (\dot{x}(t))}{\psi (x(t))}}$$

#### 2.2.2. Demodulation Analysis of the Bearing Fault Characteristic Signal via the Energy Separation Algorithm

##### Amplitude Demodulation

When a fault occurs in the inner race or rolling element, the amplitude demodulation of Equation (1), also known as the amplitude envelope, can be calculated by the energy separation algorithm as

(14) $$a(t)=C[1+\cos(2\pi f_{r}t)]\sum_{i=0}^{I}A_{k}e^{-\beta (t-iT_{p}-\tau_{i})}u(t-iT_{p}-\tau_{i})$$

By applying Fourier transform to this amplitude envelope signal, the envelope spectrum of the original signal, that is, the spectrum of the AM component, can be obtained. In the envelope spectrum, the peak frequencies will appear at the locations of fault-related frequencies, such as the fault characteristic frequency $f_{c}$ of the inner race or rolling component and its harmonics $nf_{c}$, the rotation frequency of the shaft $f_{r}$ as well as their combination $nf_{c}\pm f_{r}$.

When a fault occurs in the bearing outer race, the amplitude envelope of Equation (1) becomes

(15) $$a(t)=C\sum_{i=0}^{I}A_{k}e^{-\beta (t-iT_{p}-\tau_{i})}u(t-iT_{p}-\tau_{i})$$

Similarly, the peak frequencies will appear at the fault characteristic frequency $f_{c}$ and its harmonics $nf_{c}$ in the envelope spectrum.

##### Frequency Demodulation

The instantaneous frequency of Equation (1) is estimated by the energy separation algorithm. Its instantaneous phase is

(16) $$\varphi_{ip}=2\pi f_{n}t-\sum_{l=1}^{L}B_{il}\sin(2\pi lf_{c}t+\varphi_{il})+\varphi_{i}$$

The instantaneous frequency can be calculated as

(17) $$f_{i}(t)=\frac{1}{2\pi }d\varphi_{ip}/dt=f_{n}-\sum_{l=1}^{L}B_{il}lf_{c}\cos(2\pi lf_{c}t+\varphi_{il})$$

By applying Fourier transform to this instantaneous frequency signal, the peak frequencies will appear at the fault characteristic frequency $f_{c}$ and its harmonics $nf_{c}$ will appear in the obtained instantaneous frequency spectrum.

The above demodulation results motivated us to confirm the bearing fault by comparing peak frequencies in the envelope spectrum and instantaneous frequency spectrum of the signal with the theoretical fault-related frequencies.

**Figure 2:**
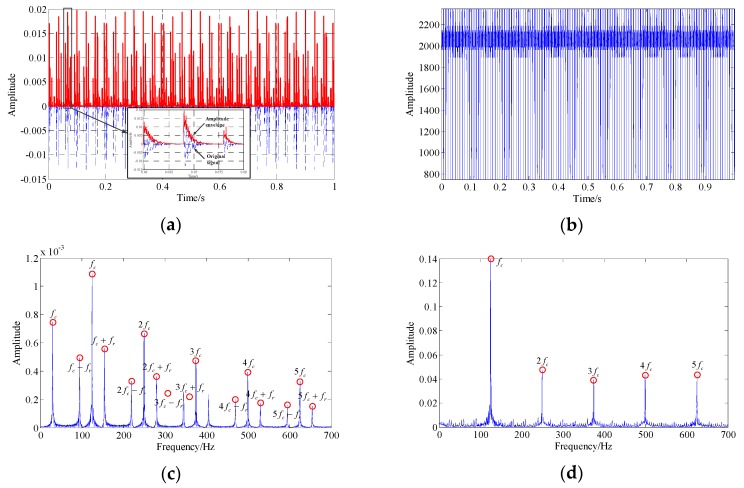
The amplitude envelope and instantaneous frequency of the signal shown in Figure 1: (**a**) amplitude envelope waveform; (**b**) instantaneous frequency waveform; (**c**) envelope spectrum; (**d**) instantaneous frequency spectrum.

Figure 2 illustrates the waveform and spectrum of the amplitude envelope and instantaneous frequency of the signal present in Figure 1, which was obtained by this energy separation algorithm. The resulting spectrum structure is much simpler than that shown in Figure 1b, especially in the instantaneous frequency spectrum where the peak frequencies appear only at the fault characteristic frequency and its harmonics. Moreover, compared with Figure 1c, the signal energy is concentrated in the low-frequency band of the envelope spectrum, which can help to highlight the fault-related frequencies located in this band from the background noise.

**Figure 3:**
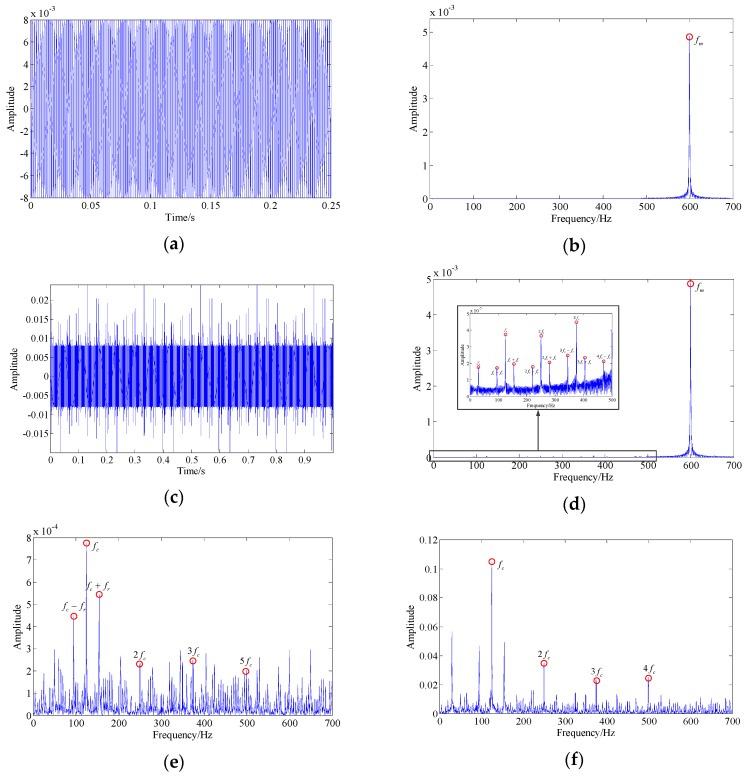
The multi-component signal consists of the signal shown in Figure 1 and the additional interference harmonic signal: (**a**,**b**) the waveform and spectrum of the added harmonic interference signal; (**c,d**) the waveform and spectrum of the multi-component signal; (**e,f**) the envelope spectrum and instantaneous frequency spectrum of the multi-component signal.

However, this algorithm can only handle single-component signals, while measured bearing vibration signals are generally composed of multiple components. Especially in the early stages of bearing fault development, the intensity of interference vibration caused by other mechanical components, such as gear meshing, compared with the weak bearing fault vibration, is usually substantial. In order to study the influence of interference components on the demodulation results of this algorithm, a low-frequency ($f_{m}=600$ Hz), large-amplitude, single-harmonic signal was added to the signal shown in Figure 1. Figure 3a,b show the waveform and spectrum of this harmonic signal. The waveform and spectrum of this multi-component signal are shown in Figure 3c,d. The fault-related frequencies (see the close-up view) are almost invisible. Figure 3e,f illustrate the envelope spectrum and instantaneous frequency spectrum of this multi-component signal. It can be observed that there are a lot of unexplainable spectral components in the spectrum, which interfere with the discrimination of fault-related frequencies.

**Figure 4:**
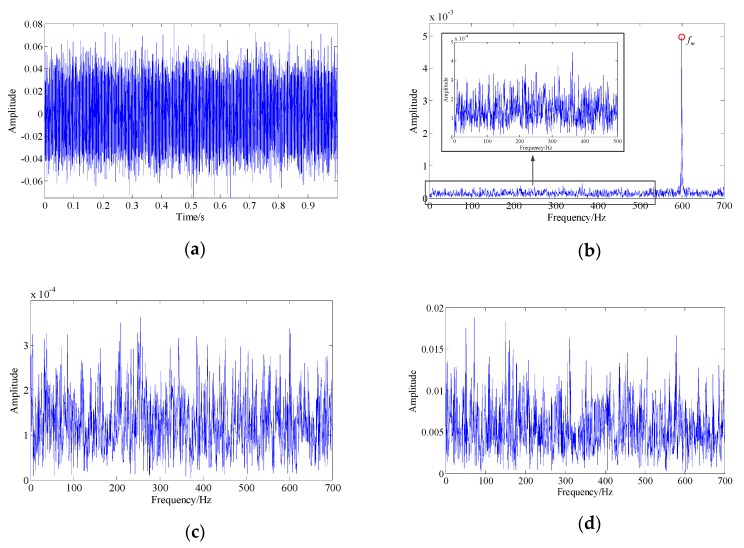
The mixed signal consists of the signal shown in Figure 3 and white Gaussian noise (signal-to-noise ratio (SNR) = -10 db): (**a**,**b**) the waveform and spectrum of the mixed signal; (**c,d**) the envelope spectrum and instantaneous frequency spectrum of the mixed signal.

Additionally, although the energy separation algorithm has a good suppressive effect on lower-energy noise, the measured vibration signal contains massive and powerful noise due to the poor working environment. In the early stages of bearing fault development, the fault characteristic signal is easily drowned out by noise. Figure 4 shows the demodulation results of a simulated noise–signal mixture based on the energy separation algorithm. This mixture consists of the multi-component signal shown in Figure 3 and strong white Gaussian noise (SNR = -10 db). Obviously, the fault-related frequency contents are completely covered by the noise spectrum and interference spectrum. As such, the existence of interference components and noise greatly affect the demodulation effect. Therefore, the suppression of background noise and the extraction of the single fault characteristic component signal must be solved before using this algorithm. To tackle this problem, we proposed a new LLORMA-based signal decomposition method.

## 3. A Novel Demodulation Analysis Technique via Energy Separation and Local Low-Rank Matrix Approximation (LLORMA)

### 3.1. Decomposing the Signal into Single Components via LLORMA

#### 3.1.1. Problem Statement and Fundamental Assumption

Problem statement: In addition to background noise, the measured one-dimensional bearing fault signal $x\in {\mathbb{R}}^{n_{1}}$ contains multiple characteristic components, such as the fault characteristic component and the unwanted interfering components. So, our mission was to suppress the strong background noise and decompose the signal into several single components to meet the requirements of the single component of the energy separation algorithm.

We transformed x into an attractor matrix $X\in {\mathbb{R}}^{n_{1}\times n_{2}}$ through phase space reconstruction to reveal the dynamic properties of the original system. This is an embedding procedure with the embedding dimension $n_{1}$ and delay time τ (where $(n_{1}-1)\tau +n_{2}=n$):

(18) $$X=\left[\begin{array}{cccc} x_{1} & x_{2} & \cdots & x_{n_{2}}\\ x_{1+\tau } & x_{2+\tau } & \cdots & x_{n_{2}+\tau }\\ \vdots & \vdots & \cdots & \vdots \\ x_{1+(n_{1}-1)\tau } & x_{2+(n_{1}-1)\tau } & \ddots & x_{n} \end{array}\right]$$

**Figure 5:**
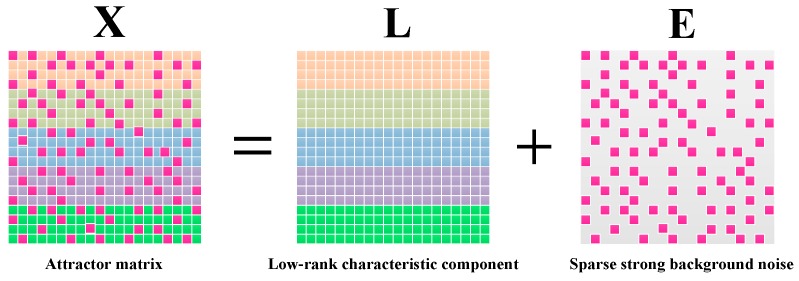
Illustration of the low rank and sparse component decomposition from the attractor matrix by RPCA.

Apparently, X is composed of multiple characteristic components and background noise. It is generally accepted that the characteristic component of matrix data is distributed on a low-dimensional sub-manifold in the high-dimensional phase space. RPCA is a commonly used method of dimension reduction analysis, which supports the characteristic component having a low rank structure [27]. Meanwhile, the noise can be suppressed by constructing a penalty function regularization. Videlicet, RPCA can robustly separate X into a low-rank component $L\in {\mathbb{R}}^{n_{1}\times n_{2}},\;r(L)\ll r(X)$ and a sparse component $E\in {\mathbb{R}}^{n_{1}\times n_{2}}$ by solving the following regularized rank minimization model (shown as Figure 5):

(19) $$\underset{L,E}{\min\arg}rank(L)+\lambda {\left\|E\right\|}_{0},\;s.t.\;X=L+E$$

where $\lambda =1/\sqrt{\max(n_{1},n_{2})}$ is a regularization parameter and $l_{0}$-norm regularization ${\left\|E\right\|}_{0}$ is the sum of the non-zero elements of E. In the field of fault feature extraction, L represents the signal characteristic component, and E captures the strong background noise. RPCA can provide a strong suppression effect on the noise, but this method is only suitable for the extraction of a single characteristic component, as shown in Equation (19). To tackle this problem, our research is based on the following fundamental assumption.

Fundamental Assumption: the characteristic component L in X is formed by multiple low-dimensional sub-manifolds, and all of them have a low rank structure.

**Figure 6:**
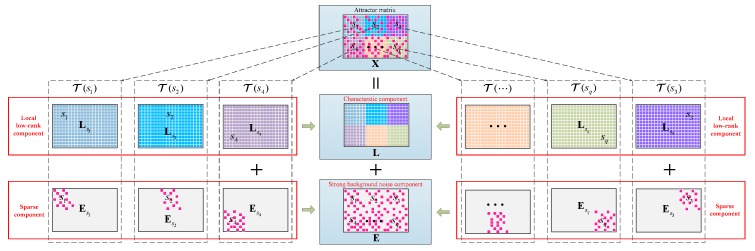
Illustration of the proposed signal decomposition method based on local low-rank matrix approximation (LLORMA).

More specifically, as shown in Figure 6, we considered that for some local selected anchor point ($s_{i}=(a_{i},b_{i}),\;a_{i}=1,\ldots ,n_{1};\;b_{i}=1,\ldots ,n_{2};\;i=1,\ldots ,q$) in X, there exists a corresponding phase space $T(s_{i})$ derived from the weighting of X, which has a single low-dimensional sub-manifold structure:

(20) $$T(s_{i})=K_{s_{1}}⊙X$$

where $K_{s_{i}}\in {\mathbb{R}}^{n_{1}\times n_{2}}$ is a local weighting coefficient matrix and the Hadamard product C=A⊙B is denoted by C(i,j)=A(i,j)B(i,j). Then, $T(s_{1})$ can be seen as the sum of a low-rank component $L_{s_{i}}$ and a sparse component $E_{s_{i}}$. Furthermore, we assumed that these low-rank matrices $L_{s_{1}},\ldots ,L_{s_{q}}$ correspond to different characteristic components: that of the fault characteristic component and the unwanted interfering components. Thus, L can be regarded as the linear regression combination of these local low-rank matrices:

(21) $$L=W_{1}⊙L_{s_{1}}+\cdots +W_{q}⊙L_{s_{q}}$$

where $W_{i}\in {\mathbb{R}}^{n_{1}\times n_{2}}$ is a linear regression matrix. So, on the basis of eliminating the noise E, our work needed to focus on decomposing L into its respective single low-rank subspaces to extract the fault characteristic component. For this purpose, we proposed a local regularized rank minimization model as follows:

(22) $$L_{s_{i}}=\underset{L_{s_{i}}}{\min\arg}\;rank\left(L_{s_{i}}\right)+\lambda {∥E_{s_{i}}∥}_{0},\;s.t.\;T\left(s_{i}\right)=L_{s_{i}}+E_{s_{i}}$$

In fact, the RPCA can be seen as a special case of the above model when each element in $K_{s_{i}}$ is equal to one. So, this model can provide state-of-the-art denoising performance.

#### 3.1.2. Model Construction and Algorithm Solving

It is critical to determine the weight coefficient matrix $K_{s_{i}}$. To begin with, we introduced a distance function $d((a,b),(a^{\prime },b^{\prime }))$ to represent the similarity between two elements X(a,b) and $X(a^{\prime },b^{\prime })$. If the distance is relatively small, then there is a high probability that these two elements belong to the same sub-manifold space, and this probability can be represented by a weight coefficient. In our research, this distance was independently divided into two parts: the distance between two rows $d(a,a^{\prime })$ and the distance between two columns $d(b,b^{\prime })$. We used the standard incomplete singular value decomposition (SVD) $X=UV^{T}$ [36] to factorize X, and these two distances were computed via the similarity between the rows of factor matrixes U and V. Particularly, the Euclid distance [37] was adopted to measure these distances:

(23) $$d(a,a^{\prime })=\frac{{\left\|U(a,:)-U(a^{\prime },:)\right\|}_{2}}{{\left\|U(a,:)\right\|}_{2}{\left\|U(a^{\prime },:)\right\|}_{2}},\;d(b,b^{\prime })=\frac{{\left\|V(b,:)-V(b^{\prime },:)\right\|}_{2}}{{\left\|V(b,:)\right\|}_{2}{\left\|V(b^{\prime },:)\right\|}_{2}}$$

It needs to be emphasized that these distances are independent of the indices of the rows or columns. That is, the adjacent elements may not be similar, while the similar elements may be spread throughout the entire space. Based on these distances, we defined $K_{s_{i}}$. The following non-parametric smoothing Epanechnikov kernel [38] was adopted to accomplish this task:

(24) $$K((a_{1},b_{1}),(a_{2},b_{2}))=K(a_{1},a_{2})K(b_{1},b_{2})=(1-d({\left(a_{1},a_{2}\right)}^{2})(1-d{\left(b_{1},b_{2}\right)}^{2})$$

We defined $K_{s_{i}}$ with its (*i*, *j*)-element as $K(s_{i},(i,j))$. $K(s_{1},s)$ monotonically decreases with $d(s_{i},s)$, that is, if the distance between any element X(s) and the anchor element $X(s_{i})$ is relatively small, the corresponding weight coefficient $K(s_{1},s)$ will be relatively large, which means that the element X(s) has a large weight that belongs to the corresponding single sub-manifold space $T(s_{i})$. In addition, theoretically, different single sub-manifold spaces will exist for different local anchor points. In contrast, for the fundamental assumption, we assumed that X is composed of only a few single sub-manifold spaces. Fortunately, the Epanechnikov kernel changes slowly, which implies that if the distance $d(s_{i},s_{j})$ is small, $T(s_{i})$ is similar to $T(s_{j})$, and both of them may represent the same single sub-manifold space. Therefore, the Epanechnikov kernel satisfies the fundamental assumption.

Based on the above theoretical description, $T(s_{i})$ was constructed. Next, $T(s_{i})$ was decomposed via solving model (22). However, this model is hard to solve due to the discrete nature combination properties of the rank function and $l_{0}$-norm. We relaxed them into their convex envelope forms, that is, using the nuclear norm and $l_{1}$-norm to replace the rank function and $l_{0}$-norm, respectively [31,39,40,41]. Hence, we obtained the following famous convex optimization model:

(25) $$L_{s_{i}}=\underset{L_{s_{i}}}{\min\arg}\;{∥L_{s_{i}}∥}_{*}+\lambda {∥E_{s_{i}}∥}_{1},\;s.t.\;T\left(s_{i}\right)=L_{s_{i}}+E_{s_{i}}$$

We determined an optimal solution to the above model via the augmented Lagrange multiplier (ALM) [42]. Algorithm 1 describes the process of this working algorithm in detail.

Via Algorithm 1, the background noise can be effectively suppressed, and q low-rank matrices can be obtained, which may represent the fault characteristic component or the unwanted interference components. Meanwhile, there may be multiple low-rank matrices representing the same single component. Therefore, the number and choice of anchor points played extremely important roles in our research. Firstly, q should be large enough to ensure that all single components can be extracted. However, this will inevitably lead to the problem of excessive computation time. Considering that there are generally no more than three main single characteristic components in the signal, we set q as 6 in our research. Furthermore, we uniformly chose the anchor points from the element set of the attractor matrix and made the distance between any two anchor points large enough to ensure that their corresponding low-rank matrix represented the different single characteristic component in the signal.

**Algorithm 1:** solve (25) by ALM**Input:** attractor matrix $X\in {\mathbb{R}}^{n_{1}\times n_{2}}$ **Parameter:** number of anchor points: q; local weight coefficient matrix: $K\in {\mathbb{R}}^{n_{1}\times n_{2}}$; regularization parameter: $\lambda =1/\sqrt{\max(n_{1},n_{2})}$ **for all**
*i*=1:q, **parallel do**  1. select $s_{i}(a_{i},b_{i})$ uniformly in X   **for all**
*a*=1: $n_{1}$, **do**    $K(a_{i},a)=1-d^{2}(a_{i},a)$   **end for all** b=1:$n_{2}$, **do**    $K(b_{i},b)=1-d^{2}(b_{i},b)$   **end for all**
$a\in [1,n_{1}],b\in [1,n_{2}]$    $K(a,b)=K(a_{i},a)K(b_{i},b)$   **end**  **2. $T(s_{i})=K⊙X$**  **Initialize:**
$L_{s_{i}}^{0}=E_{s_{i}}^{0}=Y^{0}=0$, $\tau_{0}=1e^{3}$, $\tau_{\min}=1e^{-10}$, β=0.9, ε=1e−8  **while** not converged **do**   **3.** fix the others and update $L_{s_{i}}^{k+1}$ by    $L_{s_{i}}^{k+1}=\underset{L_{s_{i}}}{\arg\min}\;:{∥L_{s_{i}}∥}_{*}+\frac{1}{2\tau_{k}}{∥L_{s_{i}}+E_{s_{i}}^{k}-T\left(s_{i}\right)+Y^{k}∥}_{F}^{2}$   **4.** fix the others and update $S_{s_{i}}^{k+1}$ by    $E_{s_{i}}^{k+1}=\underset{E_{s_{i}}}{\arg\min}\;:\lambda {∥E_{s_{i}}∥}_{1}+\frac{1}{2\tau_{k}}{∥L_{s_{i}}^{k+1}+E_{s_{i}}-T\left(s_{i}\right)+Y^{k}∥}_{F}^{2}$   **5.** update the Lagrange multiplier Y: $Y^{k+1}=Y^{k}+T\left(s_{i}\right)-L_{s_{i}}^{k+1}-E_{s_{i}}^{k+1}$   **6.** update τ: $\tau_{k+1}=\max(\rho \tau_{k},\tau_{\min})$   **7.** check the convergence conditions:    ${\left\|L_{s_{i}}^{k+1}-L_{s_{i}}^{k}\right\|}_{\infty }\leq \varepsilon$, ${\left\|E_{s_{i}}^{k+1}-E_{s_{i}}^{k}\right\|}_{\infty }\leq \varepsilon$, ${∥L_{s_{i}}^{k+1}+E_{s_{i}}^{k+1}-T\left(s_{i}\right)∥}_{\infty }\leq \varepsilon$  **end** **end** **output:**
$L_{s_{1}},\cdots ,L_{s_{q}}$


#### 3.1.3. Identification of the Fault Characteristic Component

According to Equation (4), the main instantaneous frequency of the bearing fault characteristic signal is system resonance frequency, that is, its instantaneous frequency waveform fluctuates around the system’s resonance frequency. Figure 2d confirms this important conclusion. Moreover, the system’s resonance frequency is generally much higher than the instantaneous frequency of interference signals such as the gear meshing frequency. Through inverse phase space reconstruction, the low-rank matrices can be converted into q one-dimensional single-component signals. Thus, the low-rank matrices representing the fault characteristic component can be identified by searching for the largest main frequency value among the instantaneous frequencies of these corresponding single-components.

For convenience, suppose that the p (p<q) low-rank matrices $L_{s_{1}},\ldots ,L_{s_{p}}$ are identified. In fact, according to the above analysis, these identified low-rank matrices have local sensitivity that may only describe the fault characteristic component of the subspace corresponding to the anchor point in the signal attractor matrix. Therefore, similar to Equation (15), we used the linear regression combination of these identified low-rank matrices to construct a global approximation $L_{f}\in {\mathbb{R}}^{n_{1}\times n_{2}}$ of the fault characteristic component. Lee et al. [29] used the Nadaraya–Watson regression [32] to construct the global approximation; our research also adopted this regression model, which defines the approximation as

(26) $$L_{f}=\sum_{i=1}^{p}\frac{K_{s_{i}}}{\sum_{j=1}^{p}K_{s_{j}}}⊙L_{s_{i}}$$

Specially, substituting all q low-rank matrices into this regression model gives the estimators of L composed of all the characteristic components in the signal, which can actually be seen as eliminating the noise. Equation (20) guarantees that the low-rank matrix whose anchor point is close to an element in X contributes more to the estimated value of the element in $L_{f}$ than matrixes that are further away from it. Then, through inverse phase space reconstruction, $L_{f}$ can be transformed into a one-dimensional time series $l_{f}\in {\mathbb{R}}^{n}$, that is, the single fault characteristic signal required for demodulation analysis. This completes the mission to extract the fault characteristic signal and suppress the background noise.

### 3.2. Process of the Novel Demodulation Analysis Technique

**Figure 7:**
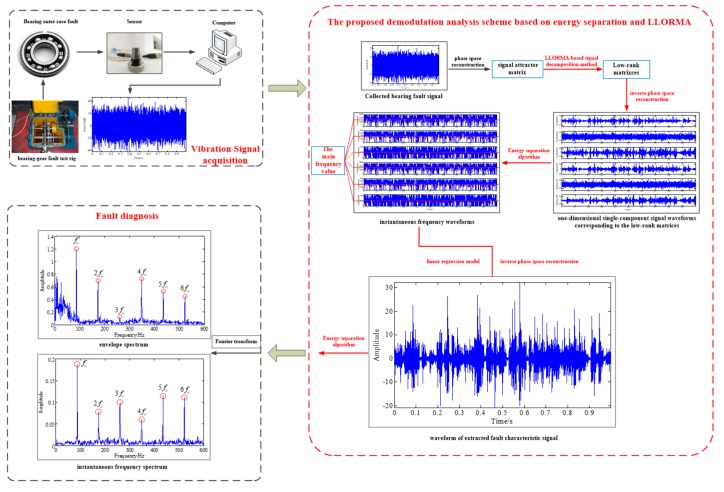
Flowchart of the proposed novel demodulation analysis technique based on energy separation and LLORMA.

To summarize the above theoretical analysis content, Figure 7 depicts a flowchart of the proposed novel demodulation analysis technique, and the key steps are listed as follows:

**Step 1:** Decompose the attractor matrix composed of the original signal into multiple low-rank matrices and, meanwhile, suppress the noise via the proposed LLORMA-based signal decomposition method;

**Step 2:** Identify the low-rank matrices representing the fault characteristic component through the high frequency characteristic of the instantaneous frequency of the modulated signal;

**Step 3:** Combine these identified low-rank matrices into a one-dimensional time series through a linear regression model and inverse phase space reconstruction to meet the demands of the single-component signal of the energy separation algorithm;

**Step 4:** Estimate the amplitude envelope and instantaneous frequency of the obtained one-dimensional time series via applying the energy separation algorithm and Fourier transform to both of them;

**Step 5:** Confirm the bearing fault by comparing the peak frequencies of the envelope spectrum and instantaneous frequency spectrum with the theoretical bearing fault-related frequencies.

## 4. Experiments

### 4.1. Numerical Simulation Analysis

In order to verify the principle and effectiveness of the proposed technique, without loss of generality, we generated a simulated noise–signal mixture, which consisted of the multi-component signal shown in Figure 3**c** and strong background noise:

(27) $$x=x_{1}(t)+x_{2}(t)+n(t)$$

where $x_{1}(t)$ is the simulated fault characteristic signal of the bearing inner race, as shown in Figure 1, and the parameters of this signal are listed in Table 1 in detail. $x_{2}(t)$ is an interference harmonic signal, as shown in Figure 3a, with a characteristic frequency of $f_{m}=600$ Hz and an amplitude of 0.008. n(t) is the background white Gaussian noise. The sampling frequency and sampling points were set as $f_{s}=$50,000 Hz and n=50,000, respectively.

The parameters of phase space reconstruction in the proposed LLORMA-based signal decomposition method were set as $n_{1}=$250 and τ=200 in accordance with the false nearest neighbor algorithm (FNN) [23]. Thus, the size of the attractor matrix was $X\in {\mathbb{R}}^{250\times 200}$.

**Figure 8:**
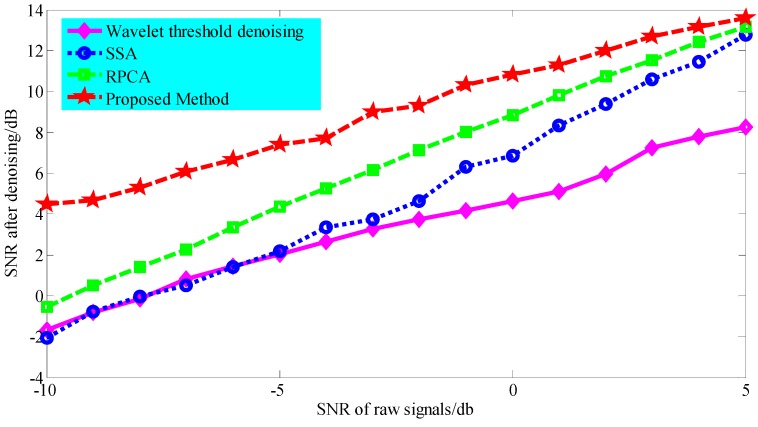
Comparison of the denoising performance of the four methods when white Gaussian noise with different SNR values was added to the multi-component signal.

We first investigated the robustness of the proposed LLORMA-based signal decomposition method to the background noise. To simulate the early stage of bearing fault development, strong background noise with an SNR ranging from –10 to 5 db was added to simulate a multi-component signal, which completely drowned out the signal characteristic information. The methods of wavelet threshold denoising, SSA, and RPCA were selected as comparative analysis methods. During wavelet threshold denoising, the decomposition layer was selected to be 3, and the wavelet basis function was set as “db15”. SSA uses the hard threshold method [43] to select the singular subspace. The parameters of RPCA are consistent with the proposed method. Figure 8 shows the SNR of the signal after denoising via the above four methods. In the vertical axis, a larger value of SNR after denoising means a better denoising performance. Obviously, the denoising performance of the proposed method is superior to that of other methods.

Then, the performance of the proposed joint demodulation technique was studied. We tested the noise–signal mixture with an SNR of –10 db, as shown in Figure 4, where the fault characteristic spectrum contents were completely submerged by interference signal and noise.

**Figure 9:**
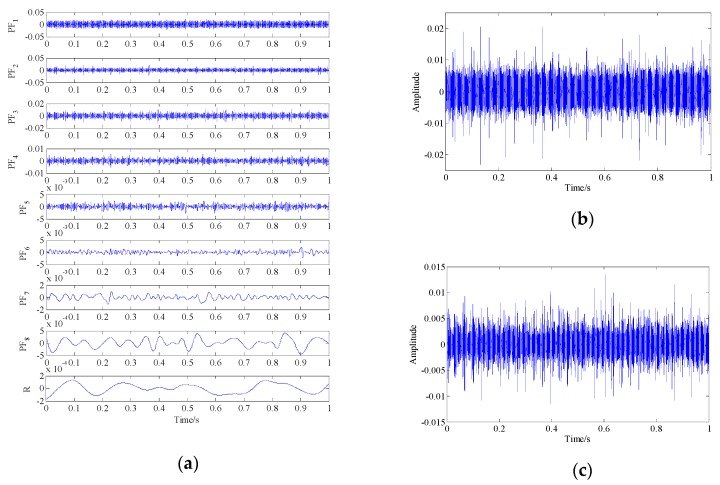

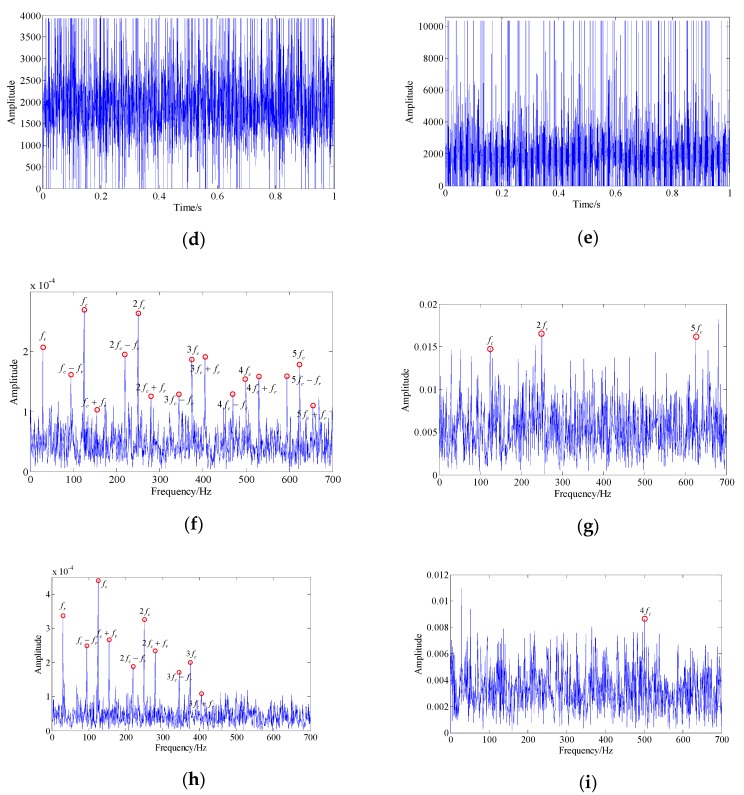
Simulation signal and analysis results determined by the demodulation analysis technique based on local mean decomposition (LMD) and energy separation: (**a**) waveform of the product functions (PFs) and residuals; (**b,c**) waveform of PF2 and PF3; (**d**,**e**) instantaneous frequency waveform of PF2 and PF3; (**f,h**) envelope spectra of PF2 and PF3; (**g,i**) instantaneous frequency spectra of PF2 and PF3.

The demodulation analysis technique based on LMD and energy separation was employed to make a comparison analysis. The waveforms of eight PF components and the residual obtained by LMD are shown as Figure 9a. By estimating the instantaneous frequency and amplitude envelope of each PF via the energy separation algorithm, it was found that the instantaneous frequency waveforms of PF2 and PF3 fluctuated around the system’s resonance frequency, as shown in Figure 9d,e. The Fourier transform was applied to the amplitude envelope and instantaneous frequency of these two PFs to obtain the envelope spectra and instantaneous frequency spectra, as shown as Figure 9f,g,h,i, respectively. For PF2, fault-related frequency peaks were found in the envelope spectrum, i.e., the rotation frequency ($f_{r}$), fault characteristic frequency ($f_{c}$), its harmonics ($2f_{c}$–$5f_{c}$) and their combinations ($f_{c}\pm f_{r}$–$5f_{c}\pm f_{r}$).$f_{c}$, and $2f_{c}$, $5f_{c}$ were found in the instantaneous frequency spectrum. For PF3, only parts of the fault-related frequency peaks appeared in the result spectrum. However, there were still many interference peaks and strong background noise in the spectrum, which seriously affected the final diagnosis result. This particularly occurred in instantaneous frequency spectra where the fault-related characteristic frequencies were hard to identify. The above discussion results demonstrate that although LMD has a certain effect on decomposing multi-component signals into single components, it cannot provide good denoising performance. Therefore, this technique was shown to be insufficient for fault diagnosis of the simulation signal in this experiment.

**Figure 10:**
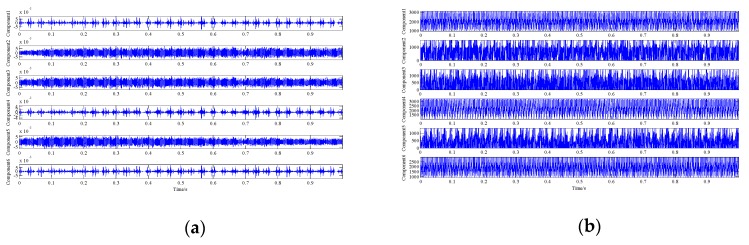

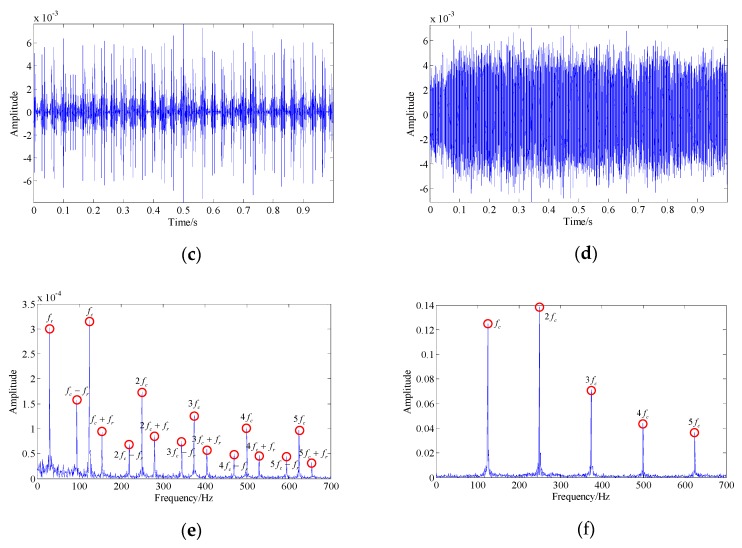
Analysis results of the proposed novel demodulation analysis technique: (**a**) waveforms of the one-dimensional single-component signals; (**b**) instantaneous frequency waveform of each component; (**c**) waveform of the extracted fault characteristic signal; (**d**) waveform of the extracted harmonic interference signal; (**e**) envelope spectrum of the extracted fault characteristic signal; **f** instantaneous frequency spectrum of the extracted signal.

We used the proposed demodulation analysis technique to address the simulation signal. Primarily, the signal was decomposed by the proposed signal decomposition method. Six low-rank matrices were obtained via Algorithm 1, and their corresponding one-dimensional single-component signals waveforms are shown in Figure 10a. Figure 10b displays the instantaneous frequency waveforms of these component signals. It can be observed that the instantaneous frequency waveforms of components 1, 4, and 6 fluctuate around the system’s resonance frequency, while the others fluctuate around the characteristic frequency of the interference harmonic signal. Therefore, we combined the low-rank matrices corresponding to components 1, 4, and 6 into a one-dimensional signal to represent the extracted fault characteristic signal, and we combined the other low-rank matrices to represent the extracted interference harmonic signal. Figure 10c,d show the waveforms of these two extracted signals, respectively. Table 2 lists the correlation coefficients between these two extracted signals and between the six one-dimensional single-component signals and the raw two characteristic signals, respectively. We can observe that the similarity between these component signals or the two extracted signals and their corresponding original characteristic signals is very high. Hence, these analysis results verify the decomposition performance of the proposed LLORMA-based signal decomposition method.

Next, the amplitude envelope and instantaneous frequency of the extracted fault characteristic signal were estimated via the energy separation algorithm, and then the Fourier transform was applied to both of them. Figure 10e,f reveal the obtained envelope spectrum and the instantaneous frequency spectrum, respectively. In the envelope spectrum, almost all of the fault-related locations appear as peaks, i.e., the rotation frequency ($f_{r}$), the fault characteristic frequency ($f_{c}$), its harmonics ($2f_{c}$–$5f_{c}$), and all of their combinations ($f_{c}\pm f_{r}$–$5f_{c}\pm f_{r}$). In the instantaneous frequency spectrum, $f_{c}$ and its harmonics ($2f_{c}$–$5f_{c}$) are very prominent. Moreover, there is almost no interference frequency peak and the background noise is suppressed at a fairly low level. So, we can confirm the fault of the simulated signal. The above simulation experiment results demonstrate that the proposed technique scheme can effectively eliminate the noise and extract the fault characteristic component from the multi-component signal, so it has an excellent diagnostic performance for the simulated signal.

### 4.2. Experimental Signal Analysis

**Figure 11:**
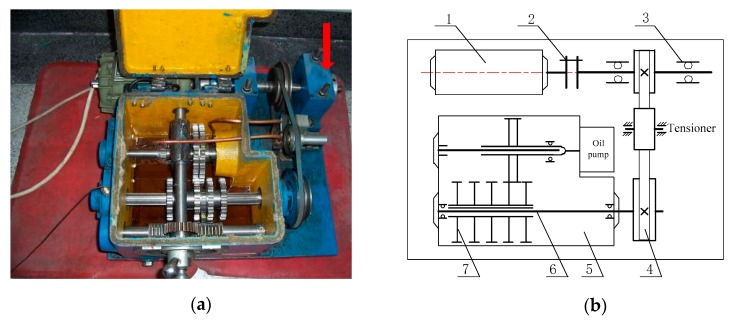
Bearing-gear fault test rig: (**a**) physical diagram; (**b**) structural diagram (1—AC motor; 2—coupler; 3—faulty bearing; 4—belt pulley; 5—gearbox; 6—transmission shaft; 7—gear sets).

In order to further verify the effectiveness of the proposed novel demodulation technique for the measured signal, we analyzed a pitting fault signal of the bearing outer race collected from a bearing-gear fault test rig. The test rig was driven by an AC motor, which is shown in Figure 11. The bearing used in this experiment was a deep groove ball bearing with the model number 6207, and the fault was caused by the electric discharge machining (EDM) method. The faulty bearing was installed at the position indicated by the red arrow in Figure 11a. We adopted the vibration acceleration sensor PCB-352C33 to collect the fault vibration signal in the vertical direction of the bearing. Table 3 lists the detailed parameters of the faulty bearing.

**Figure 12:**
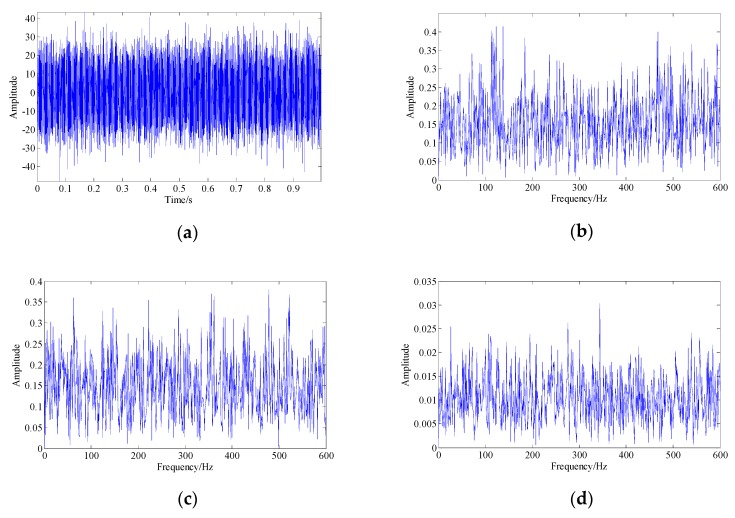
The collected pitting fault signal of the bearing outer race: (**a**) signal waveform; (**b**) signal spectrum; (**c**) envelope spectrum of the signal; (**d**) instantaneous frequency spectrum of the signal.

Figure 12 shows the waveform and spectrum of the collected fault signal as well as the spectrum of the demodulation results. Obviously, the bearing fault signal was completely drowned by the strong background noise and other interfering components, so that it could not be recognized.

**Figure 13:**
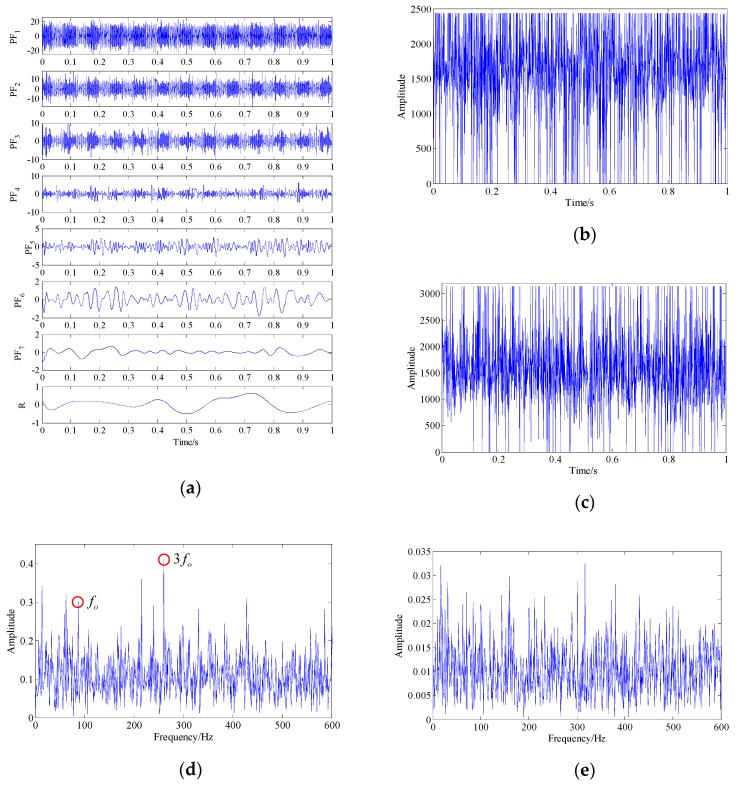

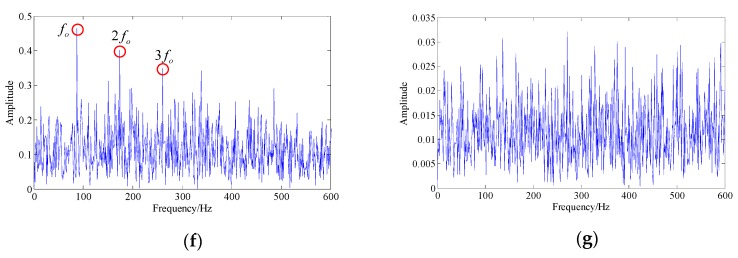
Analysis results of the demodulation analysis technique based on local mean decomposition (LMD) and energy separation: (**a**) waveform of the PFs and residuals; (**b**,**c**) instantaneous frequency waveforms of PF1 and PF2; (**d**,**f**) envelope spectra of PF1 and PF2; (**e**,**g**) instantaneous frequency spectra of PF1 and PF2**.**

We also used the demodulation analysis technique based on LMD and energy separation for the comparative analysis. The signal was decomposed into 7 PFs via LMD, and the waveforms of each PF and residual are shown in Figure 13a. Then, the amplitude envelope and instantaneous frequency of each IMF were estimated via the energy separation algorithm. We found that the instantaneous frequencies of PF1 and PF2 shown in Figure 13b,c have the largest main frequencies with a value of about 1800 Hz. Figure 13d,e,f and g show the envelope spectra and instantaneous frequency spectra of these two PFs, respectively. In the spectra shown in the demodulation results, although peaks appear at the locations of some fault-related frequencies, such as the fault characteristic frequency ($f_{o}$) and its double frequency ($2f_{o}$) and triple frequency ($3f_{o}$), the identification of this fault feature information is still difficult due to the large number of interference frequency peaks and the presence of strong background noise. So, it cannot directly be determined as to whether the bearing is faulty. These analysis results indicate that this demodulation analysis technique is not suitable for the diagnosis of the bearing fault signal in this experiment.

**Figure 14:**
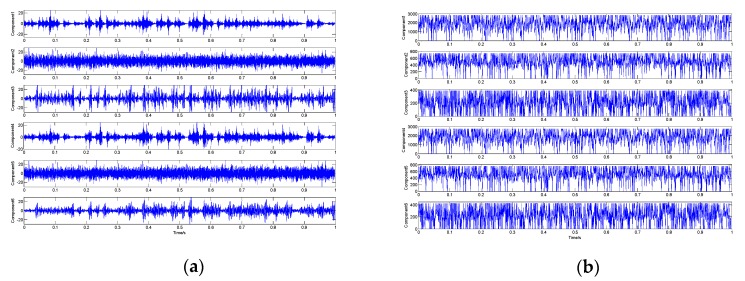

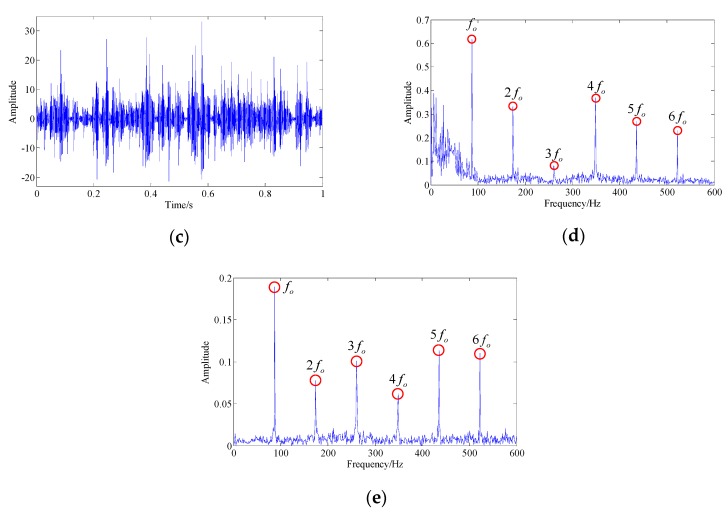
Analysis results of the proposed novel demodulation analysis technique: (**a**) waveforms of the one-dimensional single-component signals; (**b**) instantaneous frequency waveforms of each component; (**c**) waveforms of extracted fault characteristic signal; (**d**) envelope spectrum of the extracted signal; (**f**) instantaneous frequency spectrum of the extracted signal.

We then used the proposed novel demodulation analysis technique to diagnose the signal. Figure 14a shows the waveforms of the one-dimensional single-component signals corresponding to the six low-rank matrices obtained by the proposed signal decomposition method, and their instantaneous frequency waveforms are shown in Figure 14b. It can be seen that the instantaneous frequencies of components 1 and 4 have the largest main frequency values. Therefore, we combined the low-rank matrices corresponding to these two components into a one-dimensional signal via the linear regression model and inverse phase space reconstruction, which represented the fault characteristic, and the signal waveform is shown in Figure 14c. The amplitude envelope and instantaneous frequency of this signal were estimated by the energy separation algorithm, and their envelope spectrum and instantaneous frequency spectrum are shown in Figure 14d,e. In these two spectra, the positions of fault characteristic frequency ($f_{o}$) and its first few harmonics ($2f_{o}$–$6f_{o}$) all appear as peaks. In addition, we can observe that there are very few interference frequency peaks, and the background noise is almost negligible. Consequently, we can clearly determine that damage occurred in the bearing outer race. These experiment analysis results verify the effectiveness of the proposed technique in detecting and locating the fault of the collected bearing signal.

## 5. Conclusions and Future Work

The bearing vibration signal usually has modulation characteristics, and the composition of this class of signal generally includes multiple components and strong background noise. Considering the above issues, a novel demodulation analysis technique based on energy separation and LLORMA was proposed. The multi-component signal was decomposed into multiple single-component signals, and noise was suppressed by the proposed LLORMA-based signal decomposition method. Then, the energy separation algorithm was adopted to demodulate the extracted single-component signal representing the fault characteristic to achieve fault diagnosis. Finally, the principle and effectiveness of this technique were verified by analyzing both the simulation signal and the collected fault signal of the bearing outer race. The analysis results indicate that the proposed demodulation analysis technique can provide a superior diagnosis performance for the bearing fault signal.

The determination of component q in the proposed LLORMA-based decomposition method is based on experience. So, in future work, we will focus on determining the actual number of characteristic components in the original signal to determine the component number q. In addition, the identification of fault-related one-dimensional component signals and the final comparison of the fault-related frequency contents with the theoretical true values are all based on the “by hand” method, which has the drawback of automatic fault diagnosis. Therefore, another aspect of our future work is to determine the main frequency’s automatic identification in the instantaneous frequency of the component as well as to conduct automatic fault diagnostics on the extracted fault feature component.

## Figures and Tables

**Table 1:** Parameters in the simulated bearing inner race fault signal.

$f_{n}$	$f_{c}$	$f_{r}$	*I*	*L*	$A_{i}$	$B_{il}$	C	$\varphi_{i}$	$\varphi_{il}$	$\varphi_{il}$	$\tau_{j}$
2000 Hz	125 Hz	30 Hz	125	100	3e^−4^	$1/l^{2}$	1	0${}^{\circ }$	0${}^{\circ }$	800	0.02/$f_{c}$

**Table 2:** The correlation coefficients between extracted signals or the six one-dimensional single-component signals and the original two characteristic signals.

**Raw Signal**	**Component 1**	**Component 2**	**Component 3**	**Component 4**
$x_{1}(t)$	0.9002	0.0013	0.0010	0.8705
$x_{2}(t)$	0.00046	0.9272	0.9320	0.00056
**Raw Signal**	**Component 5**	**Component 6**	**Extracted Fault Characteristic Signal**	**Extracted Interference Harmonic Signal**
$x_{1}(t)$	0.0013	0.8986	0.8971	0.0013
$x_{2}(t)$	0.9241	0.00053	0.000509	0.9339

**Table 3:** Parameters of the faulty bearing.

Number of Roller Elements	Roller Diameter (mm)	Medium Diameter (mm)	Contact Angle	Rotation Frequency (Hz)	Sampling Frequency (Hz)	Sampling Points	Fault Frequency (Hz)
z = 9	d = 11.1	D = 53.5	$\alpha =0^{\circ }$	$f_{r}$ = 24.17	$f_{s}$ = 16,348	N = 16,356	$f_{o}$ = 87.01

## References

[1] Lv Y., Yuan R., Song G. (2016). Multivariate empirical mode decomposition and its application to fault diagnosis of rolling bearing. Mech. Syst. Sig. Process..

[2] Tang B., Song T., Li F., Deng L. (2014). Fault diagnosis for a wind turbine transmission system based on manifold learning and Shannon wavelet support vector machine. Renewable Energy.

[3] Liu H., Han M. (2014). A fault diagnosis method based on local mean decomposition and multi-scale entropy for roller bearings. Mech. Mach. Theory.

[4] Guo X., Chen L., Shen C. (2016). Hierarchical adaptive deep convolution neural network and its application to bearing fault diagnosis. Measurement.

[5] Smith W.A., Fan Z., Peng Z., Li H., Randall R.B. (2016). Optimised Spectral Kurtosis for bearing diagnostics under electromagnetic interference. Mech. Syst. Sig. Process..

[6] Xu C., Wang C., Liu W. (2016). Nonstationary Vibration Signal Analysis Using Wavelet-Based Time–Frequency Filter and Wigner–Ville Distribution. J. Vib. Acoust..

[7] Ming Y., Chen J., Dong G. (2011). Weak fault feature extraction of rolling bearing based on cyclic Wiener filter and envelope spectrum. Mech. Syst. Sig. Process.

[8] Wang D., Shen C., Peter W.T. (2013). A novel adaptive wavelet stripping algorithm for extracting the transients caused by bearing localized faults. J. Sound Vib..

[9] Wang Z., Zhang Q., Xiong J., Xiao M., Sun G., He J. (2017). Fault diagnosis of a rolling bearing using wavelet packet denoising and random forests. IEEE Sens. J..

[10] Daubechies I., Lu J., Wu H.T. (2011). Synchrosqueezed wavelet transforms: An empirical mode decomposition-like tool. Appl. Comput. Harmon. Anal..

[11] Hu Y., Tu X., Li F., Meng G. (2018). Joint high-order synchrosqueezing transform and multi-taper empirical wavelet transform for fault diagnosis of wind turbine planetary gearbox under nonstationary conditions. Sensors.

[12] Feng Z., Ma H., Zuo M.J. (2016). Amplitude and frequency demodulation analysis for fault diagnosis of planet bearings. J. Sound Vib..

[13] Wang Z., Wang J., Kou Y., Zhang J., Ning S., Zhao Z. (2017). Weak fault diagnosis of wind turbine gearboxes based on MED-LMD. Entropy.

[14] Liang M., Bozchalooi I.S. (2010). An energy operator approach to joint application of amplitude and frequency-demodulations for bearing fault detection. Mech. Syst. Sig. Process..

[15] Sabini E.P., Lorenc J.A., Henyan O., Hauenstein K.L. (2004). Bearing Defect Detection Using time Synchronous Averaging (TSA) of an Enveloped Accelerometer Signal. U.S. Patent.

[16] Osman S., Wang W. (2018). A leakage-free resonance sparse decomposition technique for bearing fault detection in gearboxes. Meas. Sci. Technol..

[17] Merainani B., Benazzouz D., Rahmoune C. (2017). Early detection of tooth crack damage in gearbox using empirical wavelet transform combined by Hilbert transform. J. Vib. Control.

[18] Zhao H., Li L. (2017). Fault diagnosis of wind turbine bearing based on variational mode decomposition and Teager energy operator. IET Renew. Power Gener..

[19] Ma J., Wu J., Wang X. (2018). Incipient fault feature extraction of rolling bearings based on the MVMD and Teager energy operator. ISA Trans..

[20] Zeng M., Yang Y., Zheng J., Cheng J. (2015). Normalized complex Teager energy operator demodulation method and its application to fault diagnosis in a rubbing rotor system. Mech. Syst. Sig. Process.

[21] Deng L., Zhao R. (2014). Fault feature extraction of a rotor system based on local mean decomposition and Teager energy kurtosis. J. Mech. Sci. Technol..

[22] Cai J. (2017). Feature extraction of rolling bearing fault signal based on local meandecomposition and Teager energy operator. Ind. Lubr. Tribol..

[23] Kennel M.B., Brown R., Abarbanel H.D. (1992). Determining embedding dimension for phase-space reconstruction using a geometrical construction. Phys. Rev. A.

[24] Yi C., Lv Y., Dang Z., Xiao H., Yu X. (2017). Quaternion singular spectrum analysis using convex optimization and its application to fault diagnosis of rolling bearing. Measurement.

[25] Vautard R., Yiou P., Ghil M. (1992). Singular-spectrum analysis: A toolkit for short, noisy chaotic signals. Phys. D: Nonlinear Phenom..

[26] Kouchaki S., Sanei S., Arbon E.L., Dijk D.J. (2015). Tensor based singular spectrum analysis for automatic scoring of sleep EEG. IEEE Trans. Neural Syst. Rehabil. Eng..

[27] He R., Hu B.G., Zheng W.S., Kong X.W. (2011). Robust principal component analysis based on maximum correntropy criterion. IEEE Trans. Image Process..

[28] Erichson N.B., Voronin S., Brunton S.L., Kutz J.N. (2019). Randomized Matrix Decompositions Using R. J. Stat. Software.

[29] Lee J., Kim S., Lebanon G., Singer Y., Bengio S. (2016). LLORMA: Local low-rank matrix approximation. The J. Mach. Learn. Res..

[30] Ge M., Lv Y., Yi C., Zhang Y., Chen X. (2018). A Joint Fault Diagnosis Scheme Based on Tensor Nuclear Norm Canonical Polyadic Decomposition and Multi-Scale Permutation Entropy for Gears. Entropy.

[31] Candès E.J., Recht B. (2009). Exact matrix completion via convex optimization. Found Comput. Math..

[32] Wand M.P., Jones M.C. (1994). Kernel Smoothing.

[33] Feng Z., Zuo M.J., Hao R., Chu F., Lee J. (2013). Ensemble empirical mode decomposition-based Teager energy spectrum for bearing fault diagnosis. J. Vib. Acoust..

[34] Randall R.B. (2003). A Stochastic Model for Simulation and Diagnostics of Rolling Element Bearings With Localized Faults. J. Vib. Acoust..

[35] Antoni J., Randall R.B. (2002). Differential diagnosis of gear and bearing faults. J. Vib. Acoust..

[36] Li C.Y., Zhu L., Bao W.Z., Jiang Y.L., Yuan C.A., Huang D.S. Convex Local Sensitive Low Rank Matrix Approximation. Proceedings of the 2017 International Joint Conference on Neural Networks (IJCNN).

[37] Zhang Z.L., Cao Z.Y., Li Y.T. (2010). Research Based on Euclid Distance with Weights of K_means Algorithm. J. Zhengzhou University (Eng. Sci.).

[38] Ramsay J.O. (1991). Kernel smoothing approaches to nonparametric item characteristic curve estimation. Psychometrika.

[39] Favaro P., Vidal R., Ravichandran A. A Closed Form Solution to Robust Subspace Estimation and Clustering. Proceedings of the Computer Vision and Pattern Recognition 2011.

[40] Candès E.J., Li X., Ma Y., Wright J. (2011). Robust principal component analysis?. J. ACM.

[41] Zhang H., Lin Z., Zhang C., Gao J. (2014). Robust latent low rank representation for subspace clustering. Neurocomputing.

[42] Lin Z., Chen M., Ma Y. (2009). The Augmented Lagrange Multiplier Method for Exact Recovery of Corrupted Low-Rank Matrices.

[43] Tanner J., Wei K. (2013). Normalized iterative hard thresholding for matrix completion. SIAM J.Sci. Comput..

